# Effects of Preconception Care and Periconception Interventions on Maternal Nutritional Status and Birth Outcomes in Low- and Middle-Income Countries: A Systematic Review

**DOI:** 10.3390/nu12030606

**Published:** 2020-02-26

**Authors:** Zohra S. Lassi, Sophie G. E. Kedzior, Wajeeha Tariq, Yamna Jadoon, Jai K. Das, Zulfiqar A. Bhutta

**Affiliations:** 1 Robinson Research Institute, Faculty of Health and Medical Sciences, the University of Adelaide, Adelaide 5005, Australia; sophie.kedzior@adelaide.edu.au; 2 Department of Pediatrics, the Aga Khan University, Karachi 74800, Pakistan; wajeeha.tariq2@gmail.com (W.T.); yamna.j6@gmail.com (Y.J.); jai.das@aku.edu (J.K.D.); zulfiqar.bhutta@sickkids.ca (Z.A.B.); 3 Centre for Global Child Health, the SickKids Hospital, Toronto, ON M5G 0A4, Canada

**Keywords:** periconception, folic acid, iron folic acid, delaying pregnancy, inter pregnancy interval, family planning, maternal health, neonatal health

## Abstract

Pregnancy in adolescence and malnutrition are common challenges in low- and middle-income countries (LMICs), and are associated with many complications and comorbidities. The preconception period is an ideal period for intervention as a preventative tactic for teenage pregnancy, and to increase micronutrient supplementation prior to conception. Over twenty databases and websites were searched and 45 randomized controlled trials (RCTs) or quasi-experimental interventions with intent to delay the age at first pregnancy (*n* = 26), to optimize inter-pregnancy intervals (*n* = 4), and supplementation of folic acid (*n* = 5) or a combination of iron and folic acid (*n* = 10) during the periconception period were included. The review found that educational interventions to delay the age at first pregnancy and optimizing inter-pregnancy intervals significantly improved the uptake of contraception use (RR = 1.71, 95% CI = 1.42–2.05; two studies, *n* = 911; I^2^ = 0%) and (RR = 2.25, 95% CI = 1.29–3.93; one study, *n* = 338), respectively. For periconceptional folic acid supplementation, the incidence of neural tube defects were reduced (RR = 0.53; 95% CI = 0.41–0.77; two studies, *n* = 248,056; I^2^ = 0%), and iron-folic acid supplementation improved the rates of anemia (RR = 0.66, 95% CI = 0.53–0.81; six studies; *n* = 3430, I^2^ = 88%), particularly when supplemented weekly and in a school setting. Notwithstanding the findings, more robust RCTs are required from LMICs to further support the evidence.

## 1. Introduction

Awareness of the possibility for influencing preconception health to maximize benefits for mothers and babies started upon the release of a ground-breaking report from the Centre for Disease Control [1]. Following this, the World Health Organization (WHO) gathered experts to discuss how preconception care could potentially have a positive impact on maternal and child health outcomes, where the experts overwhelmingly agreed with this proposal [2]. Subsequently there has been growth in the awareness of how important the preconception period is, as well as initiatives to increase the awareness and promotion of reproductive health from adolescence and beyond. 

To achieve ideal maternal, birth, and neonatal health outcomes, it is important to invest in preconception care [3]. Preconception care involves women’s health and its optimization prior to planning conception, and this strategy is consistently recognized as an important tool to improve both maternal and child health [4]. This period is the opportune time to integrate interventions relating to lifestyle factors, including nutrition, to promote health and to guarantee appropriate preparation for pregnancy. It is critical to establish preconception care early, particularly for girls living in low- and middle-income countries (LMICs) since the majority (99%) of all maternal and newborn deaths take place in LMICs [5]. Currently, there are a lack of preconception care policies and guidelines, and consequently, care is initiated upon realization of pregnancy and continues till childbirth and the postnatal period. Considering the known importance of preconception care, it is apparent that there is currently a missed opportunity in the system of care, especially for young girls entering their reproductive years and women who are not pregnant. Until their first pregnancy, their health and wellbeing receive scarce, if any, attention. Research shows that antenatal care is often too late to address the detrimental health risks and issues that may have affected the growing fetus [3].

Approximately 140 million births take place every year [6]. Adolescents aged 15–19 years are responsible for a staggering 16 million births, while girls younger than 16 years of age are responsible for an estimated 2.5 million births [7]. It is imperative to delay the age of women and girls at first pregnancy and optimize the inter-pregnancy intervals for adolescent girls, as well as provide preconception supplements of the essential micronutrients to promote a healthy pregnancy when a pregnancy occurs.

If the age at first pregnancy is optimal and appropriate intervals between pregnancies are achieved, it is possible to avoid many undesirable maternal, neonatal, and pregnancy outcomes. Delayed childbearing has been seen to be beneficial, particularly in adolescence, due to the numerous adverse outcomes associated with adolescent pregnancy, including the increased risk of preterm birth, stillbirth, small-for-gestational age, neonatal mortality, and labor- and delivery-related complications [8,9,10]. However, the evidence pertaining to prolonging inter-pregnancy intervals remains inconclusive. In a systematic review, Conde-Agudelo found that inter-pregnancy intervals of less than 6 months, compared to intervals of 18 to 23 months, were associated with increased risks of adverse effects. These included preterm birth, low birth weight, and small-for-gestational age babies [11]. Delaying the age of a mother’s first pregnancy ensures that her body has appropriately matured and optimizing pregnancy intervals allows it to regenerate and be primed for another pregnancy. This review is going to consider interventions for delaying the age of first pregnancy and to optimize birth intervals. Interventions can include health education, contraception education and distribution, and individual counselling or sex education, all of which can be executed in several settings, including population-based, community-based, school-based, and hospital/clinic-based settings. The interventions will also target distinct groups such as adolescents and be administered by health professionals or workers.

One area of preconception care that is well established is micronutrient supplementation during pregnancy, particularly for iron and folic acid. There are a multitude of interventions that target various vitamins and nutrients to improve maternal and neonatal outcomes. While one of the most widely known is folic acid, evidence shows that multivitamins and other nutrients are crucial to brain and nervous system development and influence the immune system during pregnancy, particularly the inflammatory response [12]. A review demonstrated that vitamin A supplementation during pregnancy may reduce maternal anemia in women who are likely to be vitamin A deficient; however, a concurrent reduction in maternal or newborn mortality was not seen [13]. Another review demonstrated that the risk of pre-eclampsia, low birthweight, and preterm birth may be reduced by vitamin D supplementation during pregnancy [14]. Evidence also exists that links multivitamin supplementation with iron and folic acid to reduce the risk of miscarriage [15].

While there are reviews investigating the effectiveness of various interventions on preventing teen pregnancies [16,17], they have mainly focused on randomized controlled trials (RCTs). The present review includes evidence from relevant experimental studies, as well as large-scale program evaluations, as randomization is not realistic for all settings and populations. To make certain that our review is thorough, we included non-randomized studies as contextual and supplementary evidence for the included RCTs [18]. The benefit of including these studies in systematic reviews includes the ability to demonstrate whether an intervention is applicable and effective in dissimilar populations, and allows us to explore possible interaction effects and to demonstrate long-term outcomes.

This review aimed to synthesize the current evidence on the effectiveness of preconception care interventions relating to the delayed age at first pregnancy; optimizing inter-pregnancy intervals; periconception folic acid; and periconception iron-folic acid supplementation on maternal, pregnancy, birth, and child outcomes using an approach of systematically reviewing primary studies, and meticulously appraising existing programs. This strategy enabled a comprehensive in-depth assessment of the effectiveness of these interventions to ameliorate the aforementioned outcomes. The evidence generated will be crucial to apprise both policy and programmatic decision-making in LMICs.

## 2. Materials and Methods

The protocol for this systematic review has been published with The Campbell Collaboration [19].

### 2.1. Objectives

The general objective was to assess how effective the following pre- and periconception interventions were at improving maternal nutrition, birth, and neonatal outcomes in LMICs when compared with no/standard intervention in terms of (1) interventions to delay the age at first pregnancy, (2) interventions to optimize inter-pregnancy intervals, (3) periconception folic acid supplementation, and (4) periconception iron folic acid supplementation.

### 2.2. Eligibility Criteria

#### 2.2.1. Types of Studies

Primary studies, including large-scale program evaluations, were used to assess the efficacy and/or effectiveness of interventions using RCTs or quasi-experimental designs (natural experiments, controlled before–after (CBA) studies, regression discontinuity designs, interrupted time series (ITS)). Pre–post studies that lacked a control group were excluded. The language was restricted to English.

#### 2.2.2. Types of Participants

Women of reproductive age (i.e., 10 to 49 years) were our target population. This included adolescent girls, regardless of health status, living in LMICs. The 2018 World Bank list of country economies [20] was used to classify country incomes; we consulted this document to ensure that studies found to be conducted in countries that were part of LMICs prior to 2018 were considered as such. While interventions were aimed at non-pregnant women, outcome measurements were for pregnant women, as well as for their children. For optimizing birth intervals, we considered interventions given to participants during pregnancy to optimize birth intervals for the next pregnancy.

#### 2.2.3. Types of Intervention

The following interventions targeting women of reproductive age (10–49 years), including adolescent girls (10–19 years), during the pre- and periconception period in LMICs were included:

- Interventions to delay the age at first pregnancy, such as curriculum-based sex education, abstinence alone programs, interactive computer-based interventions, etc.
  ○ Educational interventions and contraceptive promotion given to adolescents and young women at the community, school, or household level by parents, colleagues, teachers, health workers, or social workers.
- Interventions to optimize inter-pregnancy intervals, such as introducing family planning methods, abstinence alone programs, etc.
  ○ Educational interventions and contraceptive promotion given to mothers of reproductive age at the community, school, or household level by parents, colleagues, teachers, health workers, or social workers.
- Periconception folic acid supplementation.
  ○ Any folic acid supplementation given to either pubescent or menstruating women prior to conception that continued until the first trimester of pregnancy.
- Periconception iron folic acid.
  ○ Any iron folic acid supplementation given to either pubescent or menstruating women prior to conception and/or continued until the first trimester of pregnancy.

Interventions were compared to either no intervention, standard of care (based on study setting and what was applicable therein), or placebo. Interventions where folic acid and iron-folic acid were only used during pregnancy were not included. We excluded multiple micronutrient powders for point-of-use fortification of foods; fortification of staple foods, water, condiments, or seasonings with folic acid or iron; and other micronutrient- or folic-acid-containing oral contraceptives. Fortification programs were excluded as, by their nature, they are administered universally and do not allow the exact period of starting and stopping of intake to be known and therefore generate evidence for recommendation. We also excluded oral contraceptives that contain folic acid as they warrant a separate review altogether.

#### 2.2.4. Type of Outcome Measures

Primary outcomes:

- Maternal: unintended pregnancy, anemia, and iron deficiency anemia.
- Neonatal: neural tube defects, still birth, perinatal mortality, neonatal mortality, and low birth weight.

Secondary outcomes:

- Maternal: reported changes in knowledge and attitudes about the risk of unintended pregnancies, initiation of sexual intercourse, use of birth control methods, serum folate, adverse effects, adherence to folic acid or iron folic acid supplementation, abortion or miscarriage, and maternal mortality.
- Neonatal: preterm birth, small-for-gestational age, other congenital anomalies, and special care admission due to any reason.

**Figure 1:**
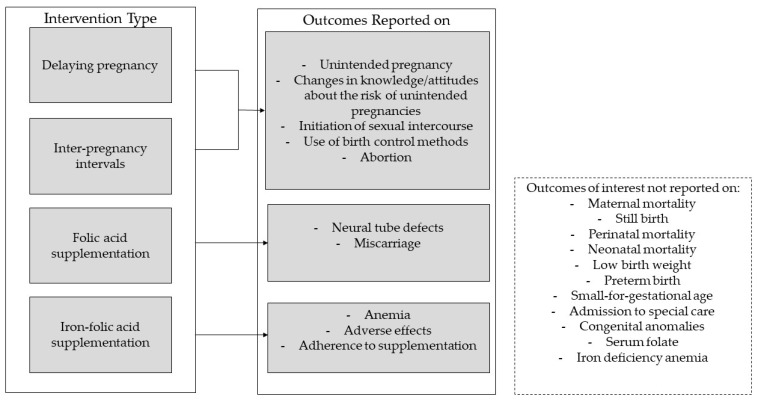
Intervention types with associated outcomes.

Studies were not included if they did not report outcomes of interests. If the outcomes were measured at any time points during pregnancy or the postpartum period, they were considered. This data was pooled based on a reasonable time point reported by most of the studies. The outcomes and their corresponding interventions are displayed in Figure 1. 

#### 2.2.5. Duration of Follow-Up

For interventions focusing on delaying the age at first pregnancy, we considered studies where the intervention was given in preconception. For interventions aiming to optimize inter-pregnancy intervals, we considered studies where interventions to optimize these intervals were given at any point in the course of the previous pregnancy, as well as interventions implemented after the birth of the last child. For folic acid supplementation, we considered studies where folic acid was supplemented during the pre- and periconception period. Lastly, for iron-folic acid supplementation, we considered studies where iron-folic acid was supplemented during the pre- and/or periconception period. 

### 2.3. Literature Search

The search was performed on May 31, 2019 using the following electronic databases: CABI’s Global Health, CINAHL, Cochrane Controlled Trials Register (CENTRAL), Dissertation Abstracts International, EMBASE, Epistemonikos, ERIC, HMIC (Health Management Information Consortium), MEDLINE, Popline, PsycINFO, Scopus, Social Science Index from Web of Science, Sociofiles, WHO’s Global Health Library, WHO Reproductive Health Library, and the WHO nutrition databases (http://www.who.int/nutrition/databases/en/). Web sites of selected development agencies or research firms (for example, JOLIS, IDEAS, IFPRI, NBER, USAID, World Bank) and Google Scholar (Appendix A) were searched.

Moreover, we checked the reference lists of all included studies and systematic reviews for further references. We tried to contact pertinent organizations and field experts to locate relevant unpublished or ongoing studies. We also scanned the references of included articles, applicable reviews, and annotated bibliographies for eligible studies.

### 2.4. Data Collection and Analysis

Data collection and analysis was carried out in conformity with the Cochrane Handbook for Systematic Reviews of Interventions [21]. 

Two review authors (Y.J. and W.T.) extracted data separately and an additional review author (Z.S.L and J.K.D.) was in place to check the data to ensure reliability and to resolve any conflict. Data for the study characteristics, including population details, setting, socio-demographic characteristics, interventions, comparators, outcomes, and study design, was extracted in duplicate. Primary study data was inspected for accuracy. Any disagreements that occurred were rectified through discourse with a third reviewer.

Once all the references were retrieved, two review authors (Z.S.L. and S.G.E.K.) independently screened their titles and abstracts. When at least one review author considered a citation possibly relevant, its full-text study report was retrieved. Two review authors (Z.S.L. and S.G.E.K.) independently screened the full text articles and identified studies for inclusion. They used a “characteristics of excluded studies” table to record the reasoning behind the exclusion of omitted studies. We resolved any disagreement through discussion or consultation with a third review author, if needed. We discovered and excluded duplicates, and to ensure that each study itself is the unit of interest, as opposed to each report, we collated various reports from the same study. We documented the entire detailed selection process to complete a Preferred Reporting Items for Systematic Reviews and Meta-Analyses (PRISMA) flow diagram [22]. 

Using a data extraction sheet, two review authors (W.T. and Y.J.) independently extracted data. We resolved any disagreement through discussion or in consultation with a third review author (Z.S.L.) if needed. We used a piloted data collection form for study characteristics and outcome data. In instances where any information was either unclear or missing, we contacted the authors of the original papers for additional details. A data extraction form was used to record data, which summarized principal characteristics of the review/studies including:

- Methods: study design and study duration.
- Details of study participants (age, socioeconomic status, parity): numbers randomized, and inclusion and exclusion criteria.
- Interventions: content, duration and timing of intervention, and comparisons.
- Outcomes and time point.

#### 2.4.1. Assessment of Risk of Bias in Included Studies

The risk of bias for each of the included studies was determined independently by two review authors (W.T. and Y.J.). We resolved any disagreements either by discussion or by consulting a third review author.

We used the Cochrane Collaboration Risk of Bias tool for all RCTs, encompassing cluster RCTs [23]. We assessed the risk of bias according to the subsequent domains. Each criterion was rated as high, low, or unclear risk.

- random sequence generation.
- allocation concealment.
- blinding of participants and personnel.
- blinding of outcome assessment for each outcome.
- incomplete outcome data.
- selective outcome reporting.
- other bias, such as validity of outcome measure and baseline comparability.

For CBA and ITS, we used Effective Practice and Organization of Care (EPOC) methods [24]. Each criterion was rated as high, low, or unclear risk.

- random sequence generation
- allocation concealment
- baseline outcome measurements
- baseline characteristics
- incomplete outcome
- knowledge of the allocated interventions adequately prevented during the study
- protection against contamination
- selective outcome reporting
- other risks of bias.

#### 2.4.2. Measures of Treatment Effect

We uploaded the outcome information for each included study into RevMan’s data tables to ascertain the treatment effects [25]. For dichotomous outcomes, we used the risk ratio (RR). For continuous outcomes reported on the same scale, we used the mean difference (MD), and for continuous outcomes that reported the same outcomes, albeit on different scales in different studies, we used the standardized mean difference (SMD). We expressed uncertainty with 95% confidence intervals (CIs) for all effect estimates. When means and standard deviations were not given, we used other obtainable data, including confidence intervals, *t*-values, *p*-values, and assigned appropriate methods described in the Cochrane Handbook for Systematic Reviews of Interventions [21] to calculate the means and standard deviations. Where other obtainable reported data was not sufficient to calculate the standard deviations, we contacted the relevant study authors. When we could not enter the results in either way, we recorded and displayed them in the Supplementary Table S1. We also considered the likelihood and implications of skewed data during analyses of continuous outcomes given that due to small sample sizes, they can provide delusive results. We also examined any relevant retraction statements and errata for information. Outcomes with multiple groups were analyzed appropriately to avoid over-counting participants by adding them to different sub-groups within the same plot. In these cases, we did not report the overall pooled estimate but instead the reported sub-group pooled estimate.

#### 2.4.3. Unit of Analysis Issues

We performed a separate meta-analysis for each topic mentioned as a separate objective. We assessed the effectiveness of each intervention as a sub-group. When trials used clustered randomization, we predicted that the investigators would have presented their results after appropriately controlling for the effects of clustering (for example, variance-inflated standard errors and hierarchical linear models). In instances where it was uncertain whether a cluster RCT had appropriately accounted for clustering, we contacted the study investigators for further information. Where appropriate controls for clustering were not made use of, we sought an estimate of the intra-class correlation coefficient (ICC). In the unlikely event that authors did not reply to our request for ICC estimates, we took the ICC reported in similar studies with a similar context. Afterward, the effect sizes and standard errors were meta-analyzed in RevMan [21]. These were merged with estimates from individual level trials.

#### 2.4.4. Dealing with Missing Data

Where possible, we contacted trial authors to confirm key study characteristics and secure absent numerical outcome data. When numerical outcome data, such as standard deviations (SDs), were not provided, and we were unable to procure these from the study authors, we calculated them from other available statistics, such as *p*-values, or using the methods outlined in the Cochrane Handbook for Systematic Reviews of Interventions [21].

#### 2.4.5. Assessment of Heterogeneity

Statistical heterogeneity was appraised using τ^2^, I^2^, and significance of the χ^2^ test. We assessed heterogeneity visually and with forest plots. Based on prior theory and clinical knowledge, we expected clinical and methodological heterogeneity in effect sizes in this literature. Therefore, we used subgroup analysis to endeavor to explain any observed statistical heterogeneity.

#### 2.4.6. Assessment of Reporting Biases

When sufficient studies were found, we drew funnel plots to explore any possible relationship between the effect size and study precision. Ten studies are usually considered sufficient to draw a funnel plot. 

#### 2.4.7. Data Synthesis

Statistical analysis was carried out using RevMan software [25]. A matrix was prepared for each intervention consisting of all the studies that outlined the dissimilarities in the studies at various levels (intervention, duration, timing, etc.), and was used to decide how best to pool the data. Random effects meta-analyses were used due to the diverse study circumstances, participants, and interventions. Findings for each comparison were descriptively summarized using contextual factors, including study setting, timings and duration of intervention, and people delivering interventions, to assess their impact on each intervention’s effectiveness.

#### 2.4.8. Assessment of Quality of Evidence

The Grading of Recommendations Assessment, Development and Evaluation (GRADE) technique was applied to individual outcomes to assess the quality of the evidence [26], which involved deliberations regarding the within-study risk of bias, directness of evidence, heterogeneity, precision of effect estimates, and risk of publication bias. “High,” “moderate,” “low,” or “very low” classifications were applied to the quality of the body of evidence for each key outcome. Non-randomized studies were rated as “low” quality at first. When there were no serious flaws in methodology, we upgraded the evidence for studies with a large magnitude of effect, presence of dose–response relationships, and the effect of plausible residual confounding. GRADE was performed for all primary outcomes.

#### 2.4.9. Subgroup Analysis and Investigation of Heterogeneity 

When there were ample studies included in the outcomes, we conducted the subgroup analyses on the following domains:

- Setting (home, facility based, community level, school, work).
- Timing of intervention (preconception, peri-conception, prenatal, post-partum).
- Type of intervention (school-based education, abstinence-only program, contraceptive promotion, etc.).

The subgroup analyses were carried out using Review Manager 5.3 with a test for interaction.

#### 2.4.10. Sensitivity Analysis 

We used sensitivity analyses to assess the potential biasing effects of using the interclass correlation coefficients.

Sensitivity analyses were planned to factor the impact of the following; however, due to the limited number of studies in each outcome, we were unable to perform these.

- Allocation concealment (adequate versus inadequate and/or unclear).
- Attrition (< 10% versus ≥ 10%).
- Imputed inter-correlation coefficients that were derived in different ways.

## 3. Results

### 3.1. Study Selection

**Figure 2:**
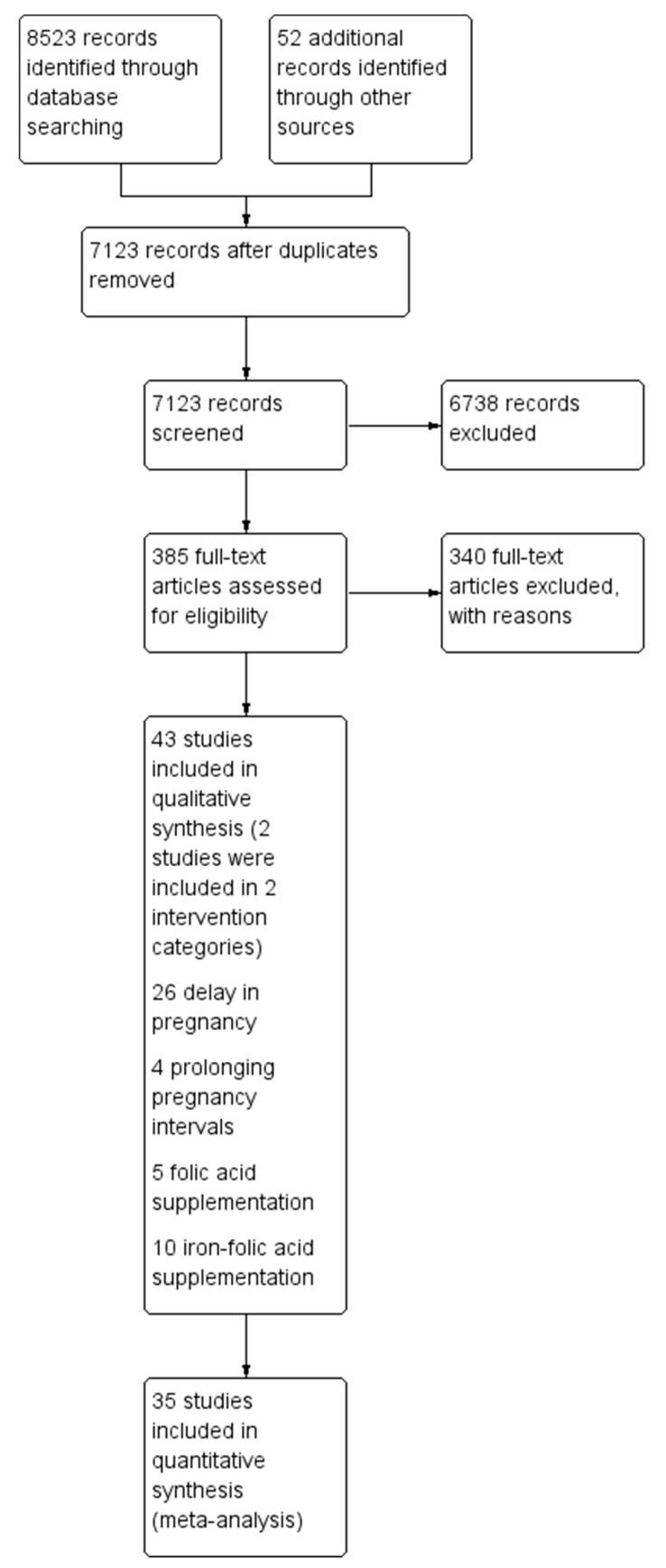
Preferred Reporting Items for Systematic Reviews and Meta-Analyses [PRISMA) flow diagram.

We identified a total of 8523 papers from the different search engines. After removing duplicates, 7123 abstracts were reviewed. Of those, 323 full texts were reviewed, and finally 45 studies were included (Figure 2). Of these, 26 were on delay in the age of pregnancy, 4 on optimizing inter-pregnancy birth intervals, 5 on the supplementation of folic acid, and 10 on the supplementation of iron-folic acid. The characteristics of the included studies are summarized in Table 1 and Supplementary Table S1. The summary of findings using the GRADE approach is summarized in Table 2, Table 3, Table 4 and Table 5.

### 3.2. Delaying Pregnancy 

#### 3.2.1. Description of Studies

A composite of 26 trials related to delaying pregnancy were included. All the trials focused on maternal outcomes, namely unintended pregnancy, reported changes in knowledge and attitudes about the risk of unintended pregnancies, initiation of sexual intercourse, use of birth control methods, and abortion.

Nineteen trials took place in Africa: one in Cameroon [50], one in Ethiopia [34], three in Kenya [32,33,36], one in Malawi [27], two in Nigeria [46], one in Senegal [31], three in South Africa [37,38,44], three in Tanzania [41,45,48], two in Uganda [49,52], one in Zimbabwe [29], and one in Zambia [70]. Five trials took place in Asia: one in China [42], three in India [30,40,47], and one in Vietnam [39]. One trial took place in South America (Chile [28]) and three trials took place in Central America: two in Mexico [35,51], and one in Belize [43].

The interventions for the trials in this review took place in varying combinations of communities, schools, and clinics. The majority of trials occurred in schools only [28,32,33,34,35,37,41,43,46,49,51,52]. One study occurred in both communities and schools [27], while seven trials had only community-based interventions [30,36,38,42,44,47,50]. Two trials had interventions occurring at community sites and in clinics [39,40]. Some trials took place in a combination of school and clinics [45], and finally, in the community, school, and clinics [29,31,48]. Regarding the sample size, the minimum population size was 366 participants in Ybarra 2013 [52] and the maximum population was 19,289 participants in Duflo [32]. The minimum included age was ten years [31,34] and the maximum was “30 years and older” [40].

Most trials investigated educational interventions, where 16 trials had education alone as the intervention [28,29,30,31,34,35,37,38,39,41,43,45,47,49,50,52]. In six trials, education was combined with other strategies: provision of contraceptives [42,44,51]; peer referrals to health care providers along with the training of health care providers [46]; training of health workers and peer condom marketing [48]; referrals, family members’ education, and improvement of contraceptive services [47]; skills training, referrals to micro savings and credit groups, and health care provider training [40]; and youth partnership group development and education subsidies [32]. Cash transfers were given to participants in the intervention group in two trials [27,36]. They were conditional in one trial [27], and unconditional in the other [36]. In one trial, there were three intervention arms: teacher training only, relative risk education only, and teacher training and relative risk education [33].

#### 3.2.2. Risk of Bias

**Figure 3:**
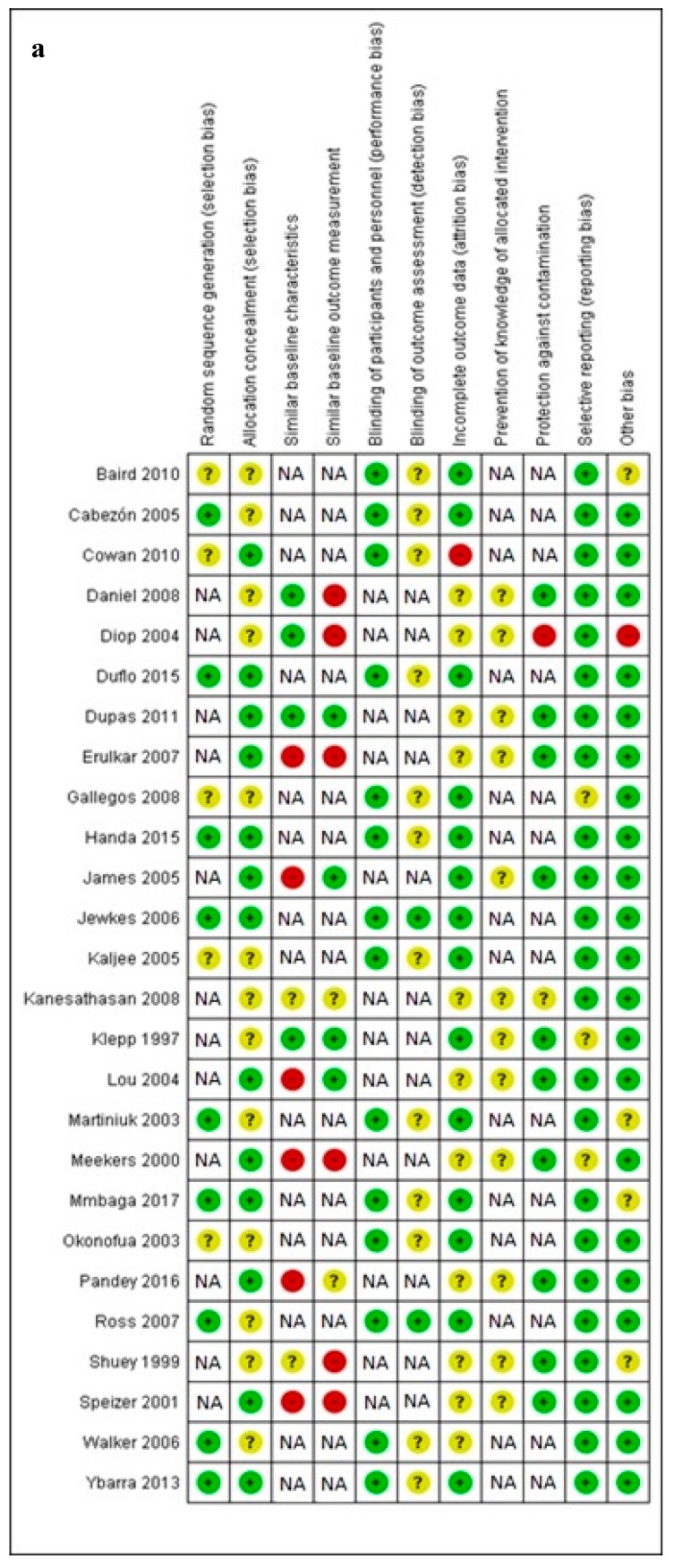

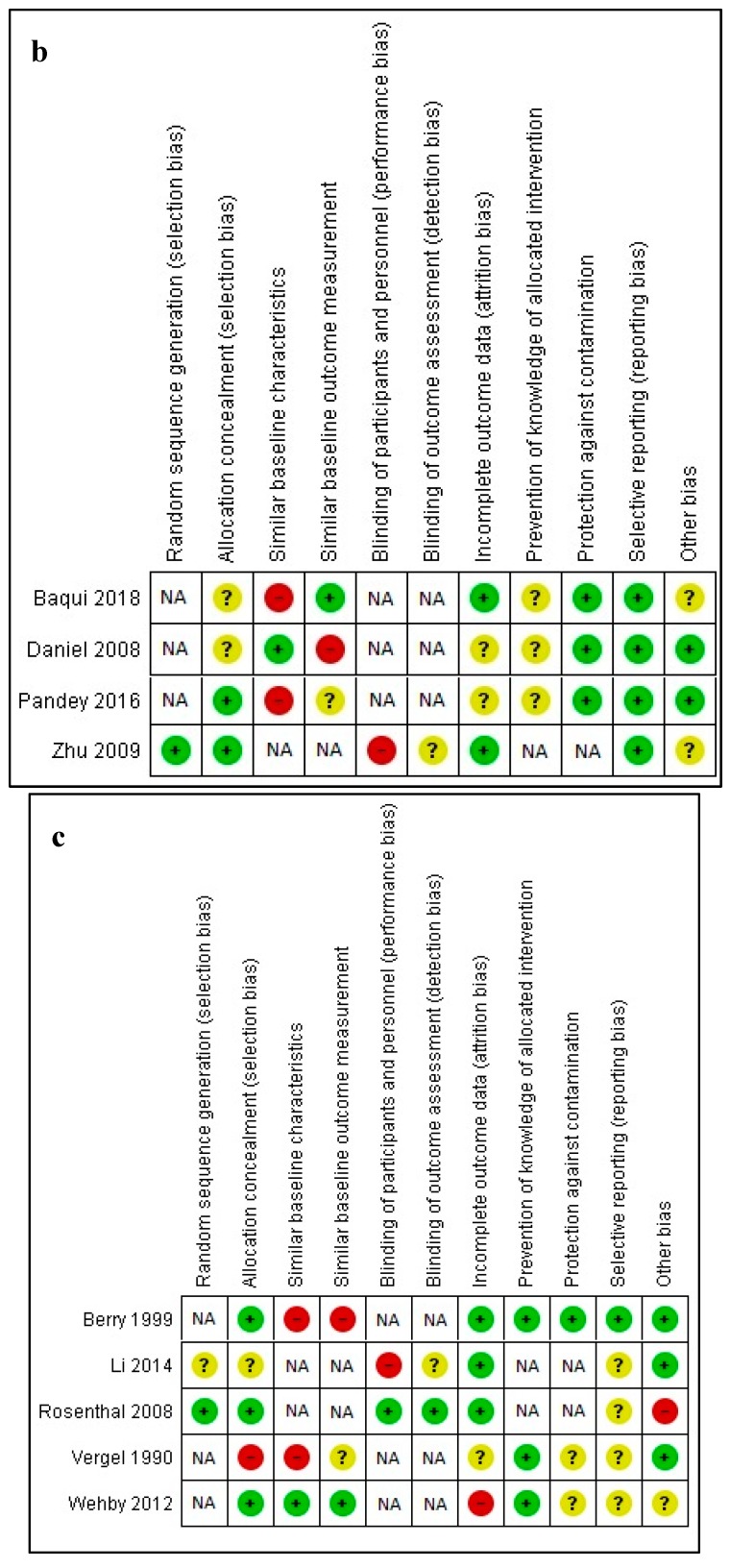

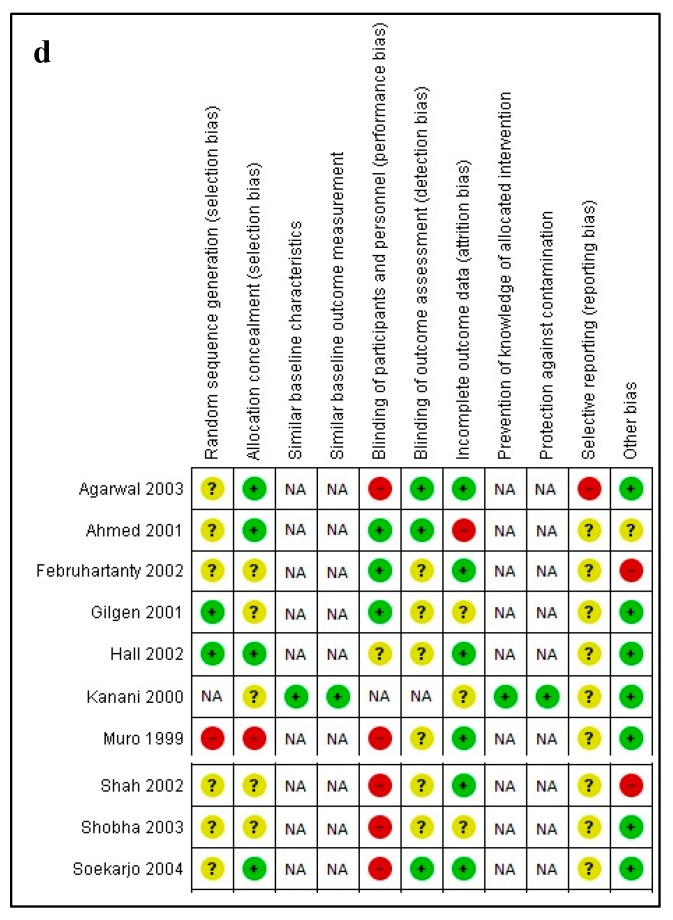
Risk of bias assessment comprised of Cochrane and EPOC criteria. Green: low risk; Red: high risk; Yellow: unclear; NA: not applicable to risk of bias due to study type. (**a**). delaying early pregnancy intervention, (**b**). prolonging inter-pregnancy intervals; (**c**): peri-conceptional iron-folic acid supplementation; (**d**). peri-conceptional folic acid supplementation.

There were a total of 14 RCTs [27,28,29,32,35,36,38,39,43,45,46,48,51,52] and the remaining were quasi-randomized studies [30,31,33,34,37,40,41,42,44,47,49,50] (Figure 3).

#### 3.2.3. Effects of Interventions 

A total of 23 studies compared education with no intervention [28,29,30,31,32,33,34,35,37,38,39,40,41,43,44,45,46,47,48,49,50,51,52]. Regarding unintended pregnancy, education on contraception did not have a significant impact on the risk of unintended pregnancy when compared with no education (RR = 0.42, 95% CI = 0.07–3.26; two studies, *n* = 490; random-effect; χ^2^ *p* = 0.009; I^2^ = 85%; low certainty of evidence using GRADE assessment). 

Two trials reported on the use of a modern birth control method, with a total of 2466 women [47,50]. One trial [50] reported the current use of modern birth control methods while the other reported whether they were ever used [47]. Education on contraception did not have a significant impact on the usage of modern methods of contraception when compared with no education (RR = 2.12, 95% CI = 0.64–7.07; two studies, *n* = 1028, random-effect, χ^2^ *p* < 0.0001, I^2^ = 94%). When this outcome was subdivided based on setting and trial type, there was no change in the risk ratio as both of the included trials had the same setting (community) and type (quasi-experimental). Eight trials [34,38,44,46,47,48,50,51] reported on the use of condoms. Education on contraception did not have a significant impact on the current usage of condoms when compared with no education (RR = 0.93, 95% CI = 0.81–1.06, eight studies, *n* = 1175, random-effect, χ^2^ *p* = 0.56; I^2^ = 0%), but it did have a significant impact on whether condoms where ever used when compared to no education (RR = 1.54, 95% CI = 1.08–2.20; six studies, *n* = 1604, random-effect, χ^2^ *p* = 0.004; I^2^ = 71%).

One trial reported on the use of a traditional birth control method, with a total of 2061 women [47]. Education on contraception did not have a significant impact on the usage of traditional methods of contraception when compared with no education (RR = 1.70, 95% CI = 0.94–3.07; one study; *n* = 623; random-effect). 

For the outcome of contraception use, three trials were included [30,34,47]. One trial [30] described current contraception use and two trials [34,47] described whether contraception was ever used. Education on contraception had a significant impact on the usage of contraception when compared with no education (RR = 2.45, 95% CI = 1.19–5.06; three studies, *n* = 2991, random-effect, heterogeneity: χ^2^ *p* < 0.00001, I^2^ = 93%). Education on contraception had a significant impact on the current use of contraception when compared with no education (RR = 4.69, 95% CI = 3.22–6.83; one study, *n* = 2080, random-effect, heterogeneity: χ^2^ *p* < 0.00001, I^2^ = 93%), as well as on ever having used contraception (RR = 1.71, 95% CI = 1.42–2.05; two studies, *n* = 911; random-effect, heterogeneity: χ^2^ *p* = 0.46; I^2^ = 0%). For ever having used contraception, the subgroups according to setting were school [34] and community [47]. Education on contraception had a significant impact on ever having used contraception when compared with no education for school (RR = 1.65, 95% CI = 1.35–2.03; one study, *n* = 288; random-effect), and community (RR = 1.95, 95% CI = 1.30–23.92; one study; *n* = 623; random-effect) settings.

One trial reported on the use of contraceptive pills [34], and demonstrated that education had a significant impact on the use of pills when compared with no education (RR = 1.34, 95% CI = 0.89–2.01; one study, *n* = 288; random-effect]. The same trial also reported on the use of injectable contraception methods [34], and this intervention had a significant impact on the use of depot/injectable methods when compared with no education (RR = 1.58, 95% CI = 1.26–1.98; one study, *n* = 288; random-effect). 

Initiation of sexual activity was described by four trials [31,45,48,52]. It was divided into three subgroups based on the time of follow up (three, six, twelve months and three years). Education on reducing sexual risks did not have a significant impact on sexual debut when compared with no education at the three-month follow-up (RR = 0.43, 95% CI = 0.04–4.51; one study, *n* = 56; random-effect), the six-month follow-up (RR = 1.02, 95% CI = 0.57–1.83; two studies, *n* = 1443, random-effect; χ^2^ *p* = 0.23, I^2^ = 29%), or at the three-year follow up (RR = 0.95, 95% CI = 0.79–1.14; two studies, *n* = 1153, random-effect; χ^2^ *p* = 0.36, I^2^ = 0%). There was a significant decrease in the age of sexual initiation at the twelve-month follow-up (RR = 0.70, 95% CI = 0.49–0.99; one study, *n* = 1387, random-effect). For the three-month follow-up outcome, there was one trial [52], which was an RCT that took place in a school setting. For the six-month follow-up outcome, there were two trials that were both RCTs [45,52], and one was a cluster RCT (cRCT) [45]. One trial took place in a school setting [52] and one took place in combined school and clinic settings [45]. For the 12-month follow-up outcome, there was one trial, which was a cRCT and took place in combined school and clinic settings [45]. For the three-year follow-up outcome, there were two trials [31,48], one was a cRCT [48], and one was a quasi RCT [31].

Seven trials reported on the changes in knowledge and attitudes about the risk of unintended pregnancies [29,30,31,43,44,47,48] and five were a part of the meta-analysis [29,30,31,44,48]. The latter included two RCTs [29,48] and three quasi RCTs [30,31,44]. Education did not have a significant impact on improving the knowledge of pregnancy prevention when compared with no education (RR = 1.02, 95% CI = 0.87–1.21; five studies, *n* = 1433, random-effect; χ^2^ *p*= 0.001; I^2^ = 78%). Education did have a significant impact on improving the knowledge of pregnancy prevention when compared with no education in RCTs (RR = 1.49, 95% CI = 1.10–2.03; two studies, *n* = 178; random-effect; χ^2^ *p* = 0.6; I^2^ = 0%), while the effect was not found to be significant in quasi RCTs (RR = 0.94, 95% CI = 0.79–1.21; three studies, *n* = 1355, random-effect; χ^2^ *p*= 0.002; I^2^ = 85%).

In Martiniuk 2003, the outcome was reported as a difference in knowledge and attitude, which was attributable to the intervention in change scores, post-test minus pre-test, between the two trial arms. For females’ knowledge, the crude score was 2.11 with a 95% CI of 0.23–3.99. For females’ attitude, the crude score was 0.05 with a 95% CI of −2.60 to 2.70 [43]. In Pandey [47], this was reported as the percentage of young women who had reported various risks to the mother or child associated with early child bearing. Almost 99.6% of young women given the intervention were aware of risks that a girl can encounter if she gives birth during adolescence at the age of 15–16 years, 38.8% were aware that underdeveloped reproductive organs lead to prolonged or obstructed labor, 50.2% were aware of the elevated likelihood of complications in pregnancy and labor/delivery, 43.0% were aware of the increased risk of maternal mortality, 6.1% were aware of miscarriage/still birth, 86.6% were aware of ill health of the mother, 1.2% were aware of anemia in women, 98.4% knew about the risks that an adolescent mother’s child may encounter, 10.5% were aware of the increased possibility of an underdeveloped child, 3.0% were aware of the increased possibility of an underdeveloped child, 4.2% were aware of the possibility of a premature birth/baby, 90.6% were aware of the risk of a weak child, 39.5% were aware of the risk of infant death, 32.0% were aware of the risk of a low birth weight (LBW) baby, 16.0% were aware of the risk of a disabled child, and 92.5% of young unmarried women had the intent to practice contraception in order to delay their first pregnancy. 

Meta-analyses were implemented for other intervention approaches too. Education plus provision of contraceptives were compared to no intervention and included one trial [42]. This approach had a significant impact on regular contraception use (RR = 1.90, 95% CI = 1.71–2.10; one study, *n* = 954; random-effect), ever having used contraception (RR = 1.17, 95% CI = 1.12–1.22; one study, *n* = 954; random-effect), and ever having used a condom (RR = 1.14, 95% CI = 1.09–1.19; one study, *n* = 954; random-effect).

Two trials also assessed the impact of conditional cash transfers. Handa 2015 reported that cash transfers to adolescent vulnerable girls reduced the possibility of pregnancy by five percentage points, while the likelihood of early marriage was not significantly impacted [36]. Whereas, Baird 2010 reported that average condom use for school girls was -0.136 (0.075) and 0.031 (0.201) post intervention [27]. 

### 3.3. Optimising Inter-Pregnancy Intervals

#### 3.3.1. Description of Studies

Four trials were included in this review relating to optimizing inter-pregnancy intervals, which involved a total of 15,718 participants. There were two quasi experimental natural experiments [30,47,53] and one randomized controlled trial [54]. The primary outcome that was reported on by the included trials was unintended pregnancy [54], other outcomes comprised of changes in knowledge and attitudes about the risk of unintended pregnancies [47], initiation of sexual intercourse [47], use of birth control methods [30,47,54], and abortion [54].

There were a total of four trials in Asia: one in Bangladesh [53], one in China [54], and two in India [30,47]. All the trials took place in community settings. The minimum population size was 2336 participants [54], and the maximum population size was 3980 participants [47]. 

Education about reproductive health and related issues, such as family planning, was the main aspect of the intervention in three trials [30,47,54,71]. There were also additional elements of behavior change communication in Daniel’s trial [30], counseling, referrals, family members’ education, and the improvement of contraceptive services in Pandey 2016 [47], as well as referrals, counseling, free provision of contraceptive materials, and involvement of the male partner in Zhu 2009 [54]. Post-partum family planning, along with maternal and newborn care for birth spacing, was the intervention in one trial [53]. 

#### 3.3.2. Risk of Bias

There was one RCT [54] and three quasi experimental studies [30,47,53] (Figure 3). 

#### 3.3.3. Effects of Interventions

For the analysis comparing education versus no intervention, two trials were included [30,47]. In Daniel’s trial [30], the change in knowledge and attitude was judged via responses to certain statements. The number of married women aged 15–24 years that agreed that early childbearing is harmful to a mother’s health increased after the intervention, from 17% to 74% in the intervention group and 12% to 65% in the control group. The number of married women aged 15–24 that agreed that contraceptive use is safe to use and required to delay first birth increased from 38% to 80% in the intervention group and 36% to 72% in the control group. In Pandey’s trial [47], 29.7% of the young married women (who had given birth at least once) in standalone areas reported the use of any modern method of contraception, compared to 18.9% in control areas. A total of 19.8% of the young married women (who had at least had one birth) in standalone areas reported the use of any modern spacing method, compared to 9.2% in control areas. 

For use of contraception, analysis of the trials [30,47] demonstrated that education on contraception did not have a significant impact on improving the use of contraception when compared with no education (RR = 2.72, 95% CI = 0.88–8.40; two studies, *n* = 2385, random-effect, heterogeneity; χ^2^ *p* < 0.0001; I^2^ = 94%), but education with the provision of contraceptives and involvement of male partner did have a significant impact (RR = 1.83, 95% CI = 1.26–2.66; one study, *n* = 338; random-effect). One trial [47], involving a total of 2061 women, reported the use of modern methods of contraception. Education on the use of modern methods of contraception when augmented with supplying contraceptives and involving the male partner had a significant impact on the use of modern methods of contraception when compared with no education (RR = 2.45, 95% CI = 1.42–4.24; one study, *n* = 338; random-effect). Subgroups according to trial setting and type were not made for this as both of the trials [30,47] under this comparison were quasi-experimental trials that took place in community settings.

Zhu [54] compared education involving the male partner with contraceptive provision with education only. Education with contraceptive provision did not have a significant impact on the risk of unintended pregnancies when compared to the less comprehensive package (RR = 0.32, 95% CI = 0.01–7.45; one study, *n* = 45; random-effect; moderate certainty of evidence using GRADE assessments). Regarding birth control methods, there was no significant impact. These included the use of any contraceptive method (RR = 1.05, 95% CI = 0.91–1.21; one study, *n* = 39; random-effect), and the use of condoms, oral contraceptives, intrauterine devices, and implants (RR = 1.08, 95% CI = 0.88–1.26; one study, *n* = 39; random-effect).

### 3.4. Periconceptional Folic Acid Supplementation

#### 3.4.1. Description of Studies

Five trials were included with a focus on folic acid, with a total of 254,746 women. The included trials mainly focused on maternal outcomes and a subset divulged outcomes related to neonatal health. There were two randomized controlled trials (RCTs) [56,57] and three quasi-experimental natural experiments [55,58,59]. The primary outcome reported was a neural tube defect. The secondary outcome reported was miscarriage.

Of the five included trials, two took place in Asia: two in China [55,56]. One trial took place in North America, in Honduras [57], and two trials took place in South America: one in Cuba [58] and one in Brazil [59]. All participants were non-pregnant women with ages ranging from 16 to 49 years, and the sample sizes ranged from 140 [57] to 247,831 [55].

Five trials [55,56,57,58,59] used periconceptional folic acid supplementation alone. Three trials supplemented women with 0.4 mg folic acid per day [55,56,59], while in the remaining trials, women were supplemented with 1 mg [57], 4 mg, and 5 mg [58] of folic acid daily. Participants were supplemented daily in all of the trials; however, one trial also had a 5 mg weekly arm [57].

#### 3.4.2. Risk of Bias

Two trials were RCTs [56,57] and the remaining three were quasi-randomized studies [55,58,59] (Figure 3).

#### 3.4.3. Effects of Interventions

Of the included trials, two [55,58] underwent a meta-analysis comparing periconceptional supplementation with folic acid versus a placebo. All these trials had daily supplementation and the dosage of folic acid was 0.4 mg [55] and 5 mg [58]. Pooled analysis found that periconceptional folic acid supplementation reduced the risk of neural tube defects (NTDs) compared to placebo by 47% (RR = 0.53, 95% CI = 0.41–0.67; three studies; *n* = 248,056; random-effect; heterogeneity: χ^2^ *p*= 0.36; I^2^ = 0%; very low certainty of evidence using GRADE assessment). However, the impact of periconceptional folic acid supplementation on NTDs was only significant for 0.4 mg of folic acid and non-significant for 5 mg of folic acid.

Miscarriage was reported in one trial [58,72]. Vergel [58] reported miscarriages as examined versus not examined. In the not-examined category, six of 124 of the unsupplemented participants and 1 of the 81 fully supplemented participants had a miscarriage. In the examined category, one of the 20 partially supplemented participants had a miscarriage (Table 1). 

### 3.5. Periconceptional Iron Folic Acid Supplementation

#### 3.5.1. Description of Studies

Ten trials investigating periconceptional iron folic acid supplementation were included with an entirety of 8955 participants. All these studies reported maternal outcomes. There were nine RCTs [60,61,62,63,64,66,67,68,69,73,74,75] and one quasi-experimental natural experiment [65]. The primary outcome reported was anemia. The secondary outcome reported was adverse effects.

Of the ten included trials, eight took place in Asia: two in Bangladesh [61,63], two in India [60,68], three in Indonesia [62,65,69], and one in Nepal [67]. Two trials took place in Africa: one in Mali [64] and one in Tanzania [66]. Seven trials were conducted in schools [60,62,64,66,67,68,69]. Soekarjo’s [69] trial also included home intervention during school holidays. Of the remaining trials, one was conducted in garment factories [61], and two in community settings [63,65,76]. Sample sizes ranged from 137 [62] to 3616 [69].

Several different dosages of iron and folic acid were used in these trials. Three studies supplemented 60 mg elemental iron and 0.25 mg folic by incorporating a weekly schedule [62,64,69], one study supplemented each of 120 mg iron and 3.5 mg folic acid [61], 100 mg iron and folate 500 µg to two groups in a daily and weekly manner [60], daily 60 mg elemental iron and 0.5 mg folic acid [65], weekly 65 mg of elemental iron with 0.25 mg folic acid [66], once daily or weekly 350 mg iron and 1.5 mg folic acid [67], daily or twice weekly 60 mg iron and 0.5 mg folic acid [68], and weekly 200 mg ferrous fumarate and 200 mg folic acid [63]. One study had a daily supplementation group [65]. Six studies had weekly arms [61,62,63,66,69]. Two studies had a daily arm and a weekly one [60,64,67]. One study had daily and twice-weekly supplementation groups [68]. All trials provided the supplementation during the preconception period. Duration of supplementation: one study supplemented for 8 weeks [66], one study supplemented for 10 weeks [64,67], seven trials provided supplementation for more than 12 weeks [60,61,62,63,65,68,69].

#### 3.5.2. Risk of Bias

There were nine RCTs [60,61,62,63,64,66,67,68,69,73,74,75] and one quasi-experimental natural experiment [65] (Figure 3).

#### 3.5.3. Effects of Interventions

Out of a total of ten trials, six presented anemia in the iron-folic acid group compared to placebo as an outcome [60,61,63,64,66,67,73,74,77]. All of them had weekly supplementation and two also had a daily supplementation group [60,67]. The trials were grouped according to type, setting, weekly or daily supplementation, and duration of weekly or daily supplementation. Overall, the analysis supports the use of iron-folic acid to reduce anemia (RR = 0.66, 95% CI = 0.53–0.81; six studies; *n* = 3430, random-effect; heterogeneity: χ^2^ *p* < 0.00001, I^2^ = 88%; very low certainty of evidence using GRADE assessment). Weekly (RR = 0.70, 95% CI = 0.55–0.88; six studies; *n* = 2661, random-effect; heterogeneity: χ^2^ *p* < 0.00001, I^2^ = 88%; very low certainty of evidence using GRADE assessments) showed a significant impact in reducing anemia compared to a placebo by 30%, while giving daily supplementation of iron-folic acid (RR = 0.49, 95% CI = 0.21–1.21; two studies; *n* = 1532, random-effect; heterogeneity: χ^2^ *p* = 0.001; I^2^ = 91%; very low certainty of evidence using GRADE assessments) had a non-significant impact. There was no significant difference between weekly iron-folic acid supplementation and a placebo when supplemented for only eight weeks (RR = 1.17, 95% CI = 0.83–1.67; one study; *n* = 159; random effect). Iron-folic acid supplementation had a significant impact at reducing anemia at school by 34% (RR = 0.66, 95% CI = 0.51–0.86; four studies; *n* = 3005, random-effect; heterogeneity: χ^2^ *p* < 0.00001, I^2^ = 87%; very low certainty of evidence using GRADE assessment) but not at work (RR = 0.59, 95% CI = 0.24–1.43; two studies; *n* = 425; random-effect; very low certainty of evidence using GRADE assessment).

Five trials reported adverse effects of iron folic acid supplementation [60,63,66,67,69], one [63,73] was meta-analyzed and found no difference in the adverse effects in the iron-folic acid supplementation group compared to the placebo group (RR = 0.63, 95% CI = 0.38–1.05; one study; *n* = 280; random-effect). Four trials examined the regularity of taking iron-folic acid supplementation regimens [61,65,66,67]. Ahmed [61] reported that, in the iron-folic acid group, the majority, i.e., 60% of participants, received 12 doses, 24% received 11 doses, 14% received 10 doses, and 2% received 9 doses. In the placebo group, 68% received 12 doses, 24% received 11 doses, 6% received 10 doses, and 2% received 9 doses. According to Kanani [65], 90% of the girls consumed greater than 85 of the 90 tablets provided. Muro [66] also reported on observed compliance to the iron-folic acid supplementation. Reported compliance was 90% in school 1, 89% in school 2, and 48% in school 3. Observed compliance was 94% in school 1, 75% in school 2, and 50% in school 3. Reported compliance was also given week-wise in all the schools. In Shah [67], eight participants (11.4%) in the group receiving daily supplementation, four (6.0%) in the weekly supplementation, and four (5.6%) in the control group were non-complaint.

## 4. Discussion

Regarding interventions aimed at delaying the age of first pregnancy, our review found three comparisons comprising of different interventions related to this intervention. Education on sexual health and contraception was the most commonly employed intervention, and the use of birth control methods was the most reported outcome. Education intervention alone showed an insignificant impact on the risk of unintended pregnancies. However, it showed a significant increase in ever having used a condom by 71%. The evidence on interventions on education and provision of contraception came from single study showing a significant impact on improving the usage of any method of contraception by 49% and the usage of condoms by 14%.

Interventions on optimizing inter-pregnancy intervals did not show a significant impact of education on and contraceptive provision along with male partner involvement on the risk of unintended pregnancies when compared to education only. However, educational intervention alone or with provision of contraceptives showed a significant improvement in the uptake of the use of contraceptives.

This review also investigated the impact of folic acid and iron-folic acid supplementation. Overall, folic acid use reduced NTD incidence by 47%. Folic acid supplementation had an impact on NTDs that differed by its dosage, as the meta-analysis showed a significant impact at 0.4 mg (reduction in incidence of NTDs by 47%) and a non-significant impact at 5 mg. For iron-folic acid, it was found that this supplementation reduced the prevalence of anemia by 34% when compared to a placebo. Weekly supplementation reduced the prevalence of anemia by 32%, while daily supplementation did not show any impact. Anemia prevalence was reduced by 34% at schools and did not show a significant impact at work when iron folic acid supplementation was given compared to a placebo. The current evidence does not support any significant difference between use of iron-folic acid and placebo to decrease adverse effects. 

The results that interventions, such as the provision of contraceptives and education to delay the age at first pregnancy, are consistent with earlier reviews [17,78,79,80,81]. Hindin included 21 studies from LMICs, which found that increased contraceptive use and delay in the age of sexual debut after interventions to prevent either unintended pregnancies or repeat pregnancies [78]. Similar to our review, Oringanje included 53 studies from LMICs and high-income countries (HICs), which also did not find evidence on reducing the risk of unintended pregnancies [17]. Similar to our review, two earlier reviews also showed that education interventions were effective at bringing about significant improvement in sexual knowledge, contraceptive use, and decreasing adolescent pregnancy and improving birth intervals [80,81].

The result that periconceptional supplementation of folic acid reduces the incidence of NTDs is consistent with other earlier reviews [12,82,83]. The results of our review on periconceptional iron-folic acid supplementation are consistent with Fernández-Gaxiola, although that review only assessed the use of iron among menstruating women (alone or with other micronutrients] in reducing the prevalence of anemia, regardless of dose used [84]. However, unlike Fernández-Gaxiola [84], our review showed that a reduction in anemia prevalence differs with the duration of iron-folic acid supplementation (no significant impact was found for supplementation for less than eight weeks).

Our review found that educational interventions that aim to delay pregnancy or optimize inter-pregnancy intervals can benefit in improving the contraceptive use and knowledge but there is insufficient evidence for the impact on unplanned pregnancy, which could be due to the shortage of included studies reporting on this outcome. Educational interventions aiming to optimize inter-pregnancy intervals were more effective than the provision of contraceptives; however, there were limited studies included in these analyses, therefore intervention strategies need to be investigated further to determine the most effective approach.

Regarding folic acid supplementation, our review further supports folic acid usage to reduce NTDs; however, the GRADE assessment determined that there was a very low certainty of evidence. Our review also favored the preconceptional use of iron-folic acid to reduce anemia, where weekly supplementation regimes were more effective. There is evidence that iron-folic acid supplementation is most effective when supplementation takes place in school settings compared to work settings, perhaps because students are more easily supervised and therefore adherence is more consistent. Therefore, our review suggests that vitamin supplementation in schools are an effective strategy to reduce anemia and that there is continuing evidence for folic acid usage to decrease NTDs although the optimal dosage is still unclear.

Further evidence is required for each of the intervention targets in this review. First, educational interventions that aim to delay pregnancy or improve inter-pregnancy intervals need to more consistently report on unplanned pregnancy. With reproductive and sexual health education often emphasizing the importance of avoiding unplanned pregnancies, studies need to ensure that these educational interventions are translating into behavior beyond contraceptive use to include the prevalence of unplanned pregnancies. Further research is required to determine whether school, community, clinical, or a combination of settings is optimal for these interventions, though there is evidence from our review that studies in community settings were more effective. A majority of the interventions had an educational component; while there was some adequate evidence from our meta-analyses of the use of educational strategies, there were fewer studies with multidimensional components (such as education and provision of contraceptives), and therefore these strategies need to be further investigated to determine whether they are just as effective or better than education alone.

Our review was consistent with other reviews investigating whether supplementing folic acid and iron-folic acid reduces the incidence of both NTDs and anemia, respectively, and therefore supplementation should be implemented before conception. Research is required to dictate the best methods to ensure consistent supplementation use of folic acid and iron-folic acid in order to determine which dosages and durations are most beneficial for maternal and neonatal outcomes. Regardless of study design, anthropometric measures should be collected when possible to ascertain the impact of micronutrient supplementation on related outcomes as this review could not report on neonatal outcomes, such as birth weight and small for gestational ageSGA. Since we included an array of study designs, it is therefore unsurprising that GRADE assessment varied from medium to very low quality.

## 5. Conclusions

While there is a growing body of evidence in support of the provision of preconception care, the effectiveness of interventions to delay the age of first pregnancy, optimize birth intervals, and to provide periconception folic acid and iron-folic acid supplementation warrant further inquiry. It is vital that we determine the most effective delivery mechanisms across different settings such that successful implementation of pre- and periconception interventions can take place in LMIC settings [85]. This review summarized and collated the present evidence for interventions executed during the pre/periconception period that aimed to delay the age at first pregnancy, optimize birth intervals, and increase the supplementation of folic and iron-folic acid with a particular focus on adolescent girls. The evidence for educational interventions focusing on delaying and optimizing intervals demonstrated a promising increase in the uptake of contraceptive use; however, no significant impact was reported for the primary outcome of unplanned pregnancies. For interventions focusing on folic acid supplementation, this review provides further evidence that this intervention in LMIC settings can successfully reduce the incidence of neural tube defects, whereas iron-folic acid supplementation can improve rates of anemia, particularly when supplemented weekly and monitored in a school setting. While we note it is important to include evidence beyond RCTs to ensure contextual factors are appropriately captured, further RCTs are required, especially for inter-pregnancy intervals and in broader LMIC locations, especially in the Americas. 

## Figures and Tables

**Table 1:** Characteristics of included studies divided by focus of intervention.

Focus of Intervention	Study	Country	Intervention	Comparison	Outcomes
Delay the age at first pregnancy	Baird et al. 2010 [27]	Malawi	Conditional cash transfer as an incentive for school girls and young women to stay or return to school	Received no conditional cash transfer	Pregnancy, initiation of sexual intercourse, condom use
	Cabezón et al. 2005 [28]	Chile	One 45-minute class per week for a year on health education, contraceptive education, skills- building, and abstinence	No intervention	Unintended pregnancy, preterm birth, spontaneous abortion
	Cowan et al. 2010 [29]	Zimbabwe	The Regai Dzive Shiri Intervention consisting of an in-school teaching program, training of nurses, raising awareness, and improving communication in the community about HIV prevention	Delayed implementation	Pregnancy
	Daniel et al. 2008 [30]	India	PRACHAR Project, which utilized a communication intervention approach	Comparison areas were chosen because their socioeconomic conditions and accessibility were similar to those of the intervention communities	Contraception use, related attitudes, and knowledge (toward early childbearing)
	Diop et al. 2004 [31]	Senegal	Three-level intervention, including community-based, clinic-based, and school-based interventions	A separate community served as the control site and did not receive any of the intervention components	Knowledge and attitudes towards reproductive health (e.g., contraception, initiation of sexual intercourse)
	Duflo et al. 2015 [32]	Kenya	Education subsidies and HIV prevention education focused on abstinence until marriage in schools; stand-alone education subsidy program	Control schools	Teenage pregnancy rate
	Dupas 2011 [33]	Kenya	Schools trained teachers for sexual and reproductive health education	Control schools did not receive any of the programs	Incidence of childbearing
	Erulkar & Methengi 2007 [34]	Ethiopia	The Berhane Hewan program with three components: (1) social mobilization and group formation by adult female mentors; (2) participation in non-formal education and livelihoods training for out of school girls, or support to remain in school; and (3) “community conversations”	A control village, selected because of its similar socioeconomic profile	Use of birth control methods, knowledge of reproductive health topics
	Gallegos et al. 2008 [35]	Mexico	Behavioral-educational intervention, which included two types of intervention: (1) reduction of HIV/AIDS risk and (2) health promotion	Control group was present, limited details were provided	Use of condoms
	Handa et al. 2015 [36]	Kenya	Monthly cash transfer to eligible households for the care and development of orphans and vulnerable residents within the household	Delayed entry into the program, due to budget constraints	Pregnancy (ever been, likelihood), initiation of sexual intercourse
	James et al. 2005 [37]	South Africa	Implementation of a photo-novella (Laduma) on knowledge, attitudes, communication, and behavioral intentions with respect to sexually transmitted infections	Did not read the photo-novella	Condom use
	Jewkes et al. 2006 [38]	South Africa	“Stepping Stones” is an HIV prevention approach that aims to improve sexual health through building stronger, more gender-equitable relationships with better communication between partners	Control arm communities attended a single session of about 3 h on HIV and safer sex	Condom use
	Kaljee et al. 2005 [39]	Vietnam	The Vietnamese Focus on Kids program, designed to teach youth new skills for decision-making and communication, as well as factual information related to reproductive health	Control youth received the intervention after collection of the 18-month follow-up data	Beliefs about condom use
	Kanesathasan et al. 2008 [40]	India	DISHA: The Development Initiative Supporting Healthy Adolescents: program, comprising of youth groups, peer education, and income generating opportunities/skills	Control sites	Knowledge and attitudes on contraception and reproductive health services; contraceptive prevalence
	Klepp et al. 1997 [41]	Tanzania	Ngao, a local HIV/AIDS education program.	Delayed-intervention comparison group	Initiation of sexual intercourse
	Lou et al. 2004 [42]	China	Community-based sex education and reproductive health service program.	Comparable site in socio-cultural, economic, and demographic characteristics; continued to provide standard program and services	Contraception use (including details of condom use)
	Martiniuk et al. 2003 [43]	Belize	Responsible sexuality education program	Classrooms were randomly allocated to the control arm	Knowledge and attitudes about the risk of unintended pregnancy
	Meekers 2000 [44]	South Africa	Targeted social marketing program and subsidized condoms	A separate control site	Knowledge and awareness of contraceptives
	Mmbaga 2017 [45]	Tanzania	PREPARE, consisting of three components implemented by teachers, peer educators, and healthcare providers (linking adolescents to information and services that may foster healthy sexuality)	Half of the primary schools were assigned to the control group based on their size and geographic location	Initiation of sexual intercourse, condom use
	Okonofua et al. 2003 [46]	Nigeria	Intervention consisted of community participation, peer education, public lectures, health clubs in the schools, and training of sexually transmitted diseases treatment providers, including those with no formal training	Randomly selected control schools that received no intervention	Condom use
	Pandey et al. 2016 [47]	India	PRACHAR Project’s reproductive health training program for adolescents, which consisted of three days of training and focused on addressing adolescents’ need for information, contraceptive supplies, parental and community support, and a youth-friendly health system	A cohort of similar young people who were not exposed to the program	Use of birth control methods (modern, condoms), knowledge and attitudes about the risk of unintended pregnancies
	Ross et al. 2007 [48]	Tanzania	Community activities; teacher-led, peer-assisted sexual health education in years 5-7 of primary school; training and supervision of health workers to provide “youth-friendly” sexual health services; and peer condom social marketing	Standard activities	Condom use, initiation of sexual intercourse
	Shuey et al. 1999 [49]	Uganda	School health education program in primary schools, consisted of nine activities involving the community, parents, local leaders, teachers, students, and school health clubs	Students in the control country area were exposed to the standard school health and AIDS education program of Uganda	Abstinence
	Speizer et al. 2001 [50]	Cameroon	Peer education program that educated peer educators in information techniques for group discussions and on reproductive health-related topics	Comparison community, Mbalmayo	Knowledge (modern contraceptives), use of birth control methods (condom use, modern contraceptives)
	Walker et al. 2006 [51]	Mexico	Two interventions: (1) HIV education, skills- building, cultural values, contraceptive promotion (condoms); and (2) HIV education, skills-building, cultural values plus contraceptive education (education and communication plus condoms and their access)	Control students received the standard biology-based sex education	Initiation of sexual intercourse, use of birth control methods (condoms, hormonal contraceptive), condom use
	Ybarra et al. 2013 [52]	Uganda	CyberSenga, a five-hour online healthy sexuality program	Received standard program (e.g., school-delivered sexuality programming)	Condom use
Optimizing inter-pregnancy intervals	Baqui et al. 2018 [53]	Uganda	Integrated post-partum family planning and maternal and newborn health interventions	Received maternal and newborn health interventions only	Preterm births, contraception use, subsequent pregnancy incidences
	Daniel et al. 2008 [30]	India	PRACHAR Project, which utilized a communication intervention approach	Comparison areas were chosen because their socioeconomic conditions and accessibility were similar to those of the intervention communities	Contraception use, related attitudes and knowledge (toward early childbearing)
	Pandey et al. 2016 [47]	India	Prachar Project’s reproductive health training program for adolescents, which consists of three days of training and focused on addressing adolescents’ need for information, contraceptive supplies, parental and community support, and a youth-friendly health system	A cohort of similar young people who were not exposed to the program	Use of birth control methods (modern, condoms), knowledge and attitudes about the risk of unintended pregnancies
	Zhu et al. 2009 [54]	China	Two post-abortion family planning (FP) service packages: (1) package included provision of limited information and referral to existing FP services, and (2) comprehensive package with additional individual counselling, free provision of contraceptive materials, and involvement of the male partner	Comparison between the two interventions	Pregnancy, repeat abortion rate, use of birth control methods
Peri-conceptional folic acid supplementation	Berry et al. 1999 [55]	China	Daily supplement containing 400 mg folic acid. Divided women who took folic acid pills according to the pattern of use based on the dates they started and stopped taking folic acid	No control, a comparison group	Neural tube defects, pregnancy outcome, pattern of use of folic acid pills
	Li et al. 2014 [56]	China	Received folic acid but did not drink milk throughout the trial	Did not take folic acid tablets and did not drink milk throughout the trial	Serum folate concentrations
	Rosenthal et al. 2008 [57]	Honduras	Two supplementation groups: (1) daily dosage of 1000 μg (1 mg) folic acid, and (2) received a once-weekly dosage of 5000 μg (5 mg)	Control	Serum folate
	Vergel et al. 1990 [58]	Cuba	5 mg folic acid/day for not less than one menstrual period before conception until the 10th week of pregnancy. (1) Fully supplemented: those who followed a full regime, and (2) partially supplemented	No supplementation, patients were in early stage of pregnancy	Pregnancy outcome (miscarriage, neural tube defects)
	Wehby et al. 2012 [59]	Brazil	Received either a single pill of 4000 μg (4 mg) folic acid or 400 μg (0.4 mg) of folic acid daily to be continued until the end of the first trimester	Historical control group	Serum folate, red blood cell folate
Peri-conceptional iron-folic acid supplementation	Agarwal et al. 2003 [60]	India	Weekly or daily iron-folate (100 mg elemental iron, 500 µg of folic)	No supplementation for first 100 days, then same as daily group	Anemia, hemoglobin concentration, plasma ferritin
	Ahmed et al. 2001 [61]	Bangladesh	Iron + folic acid (120 mg elemental Fe, 3.5 mg folic acid)	Placebo	Anemia, iron deficiency, adherence to supplementation
	Februhartanty et al. 2001 [62]	Indonesia	Two groups: (1) received a weekly iron tablet and (2) took an iron tablet for four consecutive days during their menstrual cycle. Iron tablet included 60 mg of elemental iron and 0.25 mg folic acid in the form of 200 mg ferrous sulphate	Placebo	Prevalence of anemia
	Gilgen et al. 2001 [63]	Bangladesh	Received weekly iron supplementation (200 mg ferrous fumarate and 200 mg folic acid) for 24 weeks	Placebo manufactured by the same company	Anemia
	Hall et al. 2002 [64]	Mali	Received weekly for tablets providing 65 mg iron and 0.25 mg folic acid for 10 weeks	No iron tablets were given	Prevalence of anemia, adherence to supplementation
	Kanani & Poojara 2000 [65]	India	Received iron folic acid tablets for 3 months (60 mg elemental iron + 0.5 mg folic acid per day)	Placebo supplement	Adherence to supplementation
	Muro et al. 1999 [66]	Tanzania	Iron-folic acid only (iron sulphate 65 mg and folic acid 0.25 mg)	No intervention	Anemia, adherence to supplementation, adverse effects
	Shah & Gupta2002 [67]	Nepal	Weekly vs daily iron-folic acid supplementation: (1) once daily for 90–100 days and (2) once weekly for 14 weeks; 350 mg ferrous sulfate and 1.5 mg folic acid combination	No supplementation	Anemia
	Shobha & Sharada 2003 [68]	India	Daily vs twice-weekly iron for a duration of 12 weeks; 60 mg iron, 0.5 mg folic acid	No pure control, comparison between duration	Adverse effects
	Soekarjo et al. 2004 [69]	Indonesia	Weekly 60 mg elemental iron (as ferrous sulphate) plus 250 mg folate	No supplementation	Anemia, adverse effects

**Table 2:** Delay in age of first pregnancy—Education vs. no intervention.

Education Compared to No Intervention for Delaying Pregnancy						
Patient or population: delaying at the age at first pregnancy Setting: Low- and middle-income countries (LMICs) Intervention: education Comparison: no intervention						
Outcomes	Anticipated absolute effects* (95% CI)		Relative effect (95% CI)	No. of participants (studies)	Certainty of the evidence (GRADE)	Comments
	Risk with no intervention	Risk with Education				
Unintended pregnancy	Study population		RR = 0.42 (0.07 to 2.36)	490 (2 studies)	⊕⊕⊝⊝ LOW 1 2	
	122 per 1000	132 per 1000 (41 to 420)				
*The risk in the intervention group (and its 95% confidence interval) was based on the assumed risk in the comparison group and the relative effect of the intervention (and its 95% CI). CI: confidence interval; RR: risk ratio; OR: odds ratio.						
GRADE Working Group grades of evidence: High certainty: We are very confident that the true effect lies close to that of the estimate of the effect. Moderate certainty: We are moderately confident in the effect estimate; the true effect is likely to be close to the estimate of the effect, but there is a possibility that it is substantially different. Low certainty: Our confidence in the effect estimate is limited; the true effect may be substantially different from the estimate of the effect. Very low certainty: We have very little confidence in the effect estimate; the true effect is likely to be substantially different from the estimate of effect.						

1. There is a high risk of attrition bias due to greater than 20% patients being lost before follow up from both intervention and control arms. 2. High risk of selection bias.

**Table 3:** Optimizing inter-pregnancy interval—Education + provision of contraception + involvement of male partner vs. education alone.

Education + Referral Services + Training of Service Providers + Counselling + Provision of Contraception + Involvement of Male Partner Compared to Education + Referral Services in Pregnancy						
Patient or population: pregnancy Setting: LMICs Intervention: education + referral services + training of service providers + counselling + provision of contraception + involvement of male partner Comparison: education + referral services						
Outcomes	Anticipated absolute effects* (95% CI)		Relative effect (95% CI)	No. of participants (studies)	Certainty of the evidence (GRADE)	Comments
	Risk with education + referral services	Risk with education + referral services + training of service providers + counselling + provision of contraception + involvement of male partner				
Unintended pregnancies	Study population		RR = 0.32 (0.01 to 7.45)	45 (1 randomized controlled trial (RCT))	⊕⊕⊕⊝ MODERATE 1 2	
	45 per 1000	15 per 1000 (0 to 339)				
*The risk in the intervention group (and its 95% confidence interval) is based on the assumed risk in the comparison group and the relative effect of the intervention (and its 95% CI). CI: confidence interval; RR: risk ratio; OR: odds ratio.						
GRADE Working Group grades of evidence: High certainty: We are very confident that the true effect lies close to that of the estimate of the effect.Moderate certainty: We are moderately confident in the effect estimate; the true effect is likely to be close to the estimate of the effect, but there is a possibility that it is substantially different. Low certainty: Our confidence in the effect estimate is limited; the true effect may be substantially different from the estimate of the effect. Very low certainty: We have very little confidence in the effect estimate; the true effect is likely to be substantially different from the estimate of effect.						

1. Heterogeneity not applicable as there was only one study under this comparison. 2. Total number of events was less than 300.

**Table 4:** Periconceptional folic acid supplementation compared to placebo.

Folic Acid Compared to Placebo for Periconceptional Women						
Patient or population: periconceptional womenSetting: LMICs Intervention: Folic acid Comparison: Placebo						
Outcomes	Anticipated absolute effects* (95% CI)		Relative effect (95% CI)	No. of participants (studies)	Certainty of the evidence (GRADE)	Comments
	Risk with placebo	Risk with folic acid				
Neural tube defects	Study population		RR = 0.53 (0.41 to 0.67)	248,056 (2 RCTs)	⊕⊝⊝⊝ VERY LOW 1 2	
	2 per 1000	1 per 1000 (1 to 1)				
*The risk in the intervention group (and its 95% confidence interval) was based on the assumed risk in the comparison group and the relative effect of the intervention (and its 95% CI). CI: confidence interval; RR: risk ratio; OR: odds ratio.						
GRADE Working Group grades of evidence: High certainty: We are very confident that the true effect lies close to that of the estimate of the effect. Moderate certainty: We are moderately confident in the effect estimate; the true effect is likely to be close to the estimate of the effect, but there is a possibility that it is substantially different. Low certainty: Our confidence in the effect estimate is limited; the true effect may be substantially different from the estimate of the effect. Very low certainty: We have very little confidence in the effect estimate; the true effect is likely to be substantially different from the estimate of effect.						

1. Two studies (Berry et al. 1999 [55]) (Vergel et al. 1990 [58]) did not have random sequence generation and allocation concealment. 2. Number of events was less than 300.

**Table 5:** Periconceptional iron folic acid supplementation compared to placebo.

Iron Folic Acid Compared to Placebo for Periconceptional Women						
Patient or population: periconceptional women Setting: LMICs Intervention: Iron folic acid Comparison: Placebo						
Outcomes	Anticipated absolute effects* (95% CI)		Relative effect (95% CI)	No. of participants (studies)	Certainty of the evidence (GRADE)	Comments
	Risk with placebo	Risk with iron-folic acid				
Anemia – RCTs	Study population		RR = 0.66 (0.53 to 0.81)	3430 (6 RCTs)	⊕⊝⊝⊝ VERY LOW 1 2 3	
	565 per 1000	350 per 1000 (288 to 429)				
Anemia—Weekly supplementation	Study population		RR = 0.70 (0.55 to 0.88)	2661 (6 RCTs)	⊕⊝⊝⊝ VERY LOW 1 2 6	
	488 per 1000	332 per 1000 (273 to 405)				
Anemia—Daily supplementation	Study population		RR = 0.49 (0.21 to 1.12)	1532 (2 RCTs)	⊕⊝⊝⊝ VERY LOW 1 2 7	
	417 per 1000	213 per 1000 (133 to 338)				
Anemia—8 weeks of weekly supplementation	Study population		RR = 1.17 (0.55 to 1.67)	159(1 RCTs)	⊕⊝⊝⊝ VERY LOW4 5 8	
	249 per 1000	237 per 1000 (142 to 394)				
Anemia—10 weeks of weekly supplementation	Study population		RR = 0.75 (0.64 to 0.88)	552 (1 RCT)	⊕⊕⊝⊝ VERY LOW 4 9	
	609 per 1000	456 per 1000 (389 to 536)				
Anemia—12 weeks of weekly supplementation	Study population		RR = 0.39 (0.27 to 0.57)	145 (1 RCTs)	⊕⊝⊝⊝ VERY LOW 1 2 4 7	
	398 per 1000	187 per 1000 (108 to 327)				
Anemia—14 weeks of weekly supplementation	Study population		RR = 0.21 (0.11 to 0.39)	139 (1 RCT)	⊕⊕⊝⊝ LOW 4 10	
	653 per 1000	137 per 1000 (72 to 255)				
Anemia—16 weeks of weekly supplementation	Study population		RR = 0.89 (0.79 to 0.99)	1386 (1 RCT)	⊕⊕⊕⊝ MODERATE 9	
	504 per 1000	448 per 1000 (398 to 499)				
Anemia—24 weeks of weekly supplementation	Study population		RR = 0.85 (0.77 to 0.94)	280 (1 RCT)	⊕⊕⊝⊝ LOW 4 11	
	915 per 1000	778 per 1000 (704 to 860)				
Anemia—School	Study population		RR = 0.66 (0.51to 0.86)	3005 (4 RCTs)	⊕⊝⊝⊝ VERY LOW 1 2 12	
	459 per 1000	257 per 1000 (206 to 326)				
Anemia—Work	Study population		RR = 0.59 (0.24 to 1.43)	425 (2 RCTs)	⊕⊝⊝⊝ VERY LOW 4 13 14	
	863 per 1000	509 per 1000 (207 to 1000)				
*The risk in the intervention group (and its 95% confidence interval) was based on the assumed risk in the comparison group and the relative effect of the intervention (and its 95% CI). CI: confidence interval; RR: risk ratio; OR: odds ratio.						
GRADE Working Group grades of evidence: High certainty: We are very confident that the true effect lies close to that of the estimate of the effect. Moderate certainty: We are moderately confident in the effect estimate; the true effect is likely to be close to the estimate of the effect, but there is a possibility that it is substantially different. Low certainty: Our confidence in the effect estimate is limited; the true effect may be substantially different from the estimate of the effect. Very low certainty: We have very little confidence in the effect estimate; the true effect is likely to be substantially different from the estimate of effect.						

1. Some studies use multiple micronutrients in the intervention arm. 2. Multiple studies with a large weightage are at high risk for bias. 3. Heterogeneity was 84%. 4. Total number of events was less than 300. 5. One study used vitamin C along with iron-folic acid in the intervention arm. 6. Heterogeneity was 82%. 7. Heterogeneity was 76%. 8. One study was at high risk of bias. 9. Study was at risk of performance and reporting bias. 10. Study was at risk of other biases. 11. It was mostly unclear if study was at risk of bias. 12. Heterogeneity was 83%. 13. Heterogeneity was 95%. 14. One study was at risk of attrition bias.

## Supplementary Materials

The following are available online at https://www.mdpi.com/2072-6643/12/3/606/s1, Table S1: Location the interventions were conducted by intervention type.

## Appendix A

EMBASE Search Strategy

Pubmed - Delay/Interval

[1] "Preconception Care"[Mesh] OR (("preconception"[Text Word] OR "preconception care"[Text Word] OR "periconception"[Text Word] OR "pre-pregnancy"[Text Word])) OR ("preconception"[Title/Abstract] OR "preconception care"[Title/Abstract] OR "periconception"[Title/Abstract] OR "pre-pregnancy"[Title/Abstract])

[2] (("pregnancy interval*"[Text Word] OR "birth spacing"[Text Word] OR "first birth interval"[Text Word] OR "interpregnancy interval"[Text Word] OR "intergenesic interval"[Text Word] OR "pregnancy spacing"[Text Word] OR "family planning"[Text Word] OR "delaying childbearing"[Text Word] OR "delaying pregnancy"[Text Word] OR "delay pregnancy"[Text Word]) OR ("pregnancy interval*"[Title/Abstract] OR "birth spacing"[Title/Abstract] OR "first birth interval"[Title/Abstract] OR "interpregnancy interval"[Title/Abstract] OR "intergenesic interval"[Title/Abstract] OR "pregnancy spacing"[Title/Abstract] OR "family planning"[Title/Abstract] OR "delaying childbearing"[Title/Abstract] OR "delaying pregnancy"[Title/Abstract] OR "delay pregnancy"[Title/Abstract])) OR birth intervals[MeSH Terms]

[3] ("reproductive aged women" OR "adolescen*" OR "teen*" OR women)

[1] AND [2]

[1] AND [2] AND [3]

PubMed/Medline: Folic/iron acid

[1] "Preconception Care"[Mesh] OR (("preconception"[Text Word] OR "preconception care"[Text Word] OR "periconception"[Text Word] OR "pre-pregnancy"[Text Word])) OR ("preconception"[Title/Abstract] OR "preconception care"[Title/Abstract] OR "periconception"[Title/Abstract] OR "pre-pregnancy"[Title/Abstract])

[2] (((((("iron folic acid"[Text Word]) OR "iron folic acid"[Title/Abstract]) OR "folic acid"[MeSH Major Topic]) OR "folic acid"[Text Word]) OR "folic acid"[Title/Abstract]) OR "iron folic acid supplementation") OR "folic acid supplementation"

[3] ((("reproductive aged women" OR "adolescen*" OR "teen*" OR women)) OR ((("Adolescent"[Mesh]) OR "Menstruation"[Mesh]) OR "Puberty"[Mesh]))

[1] AND [2] AND [3]

[1] AND [2] AND [3] AND NOT “pregnant”

Embase - Delay/Interval

[1] ’prepregnancy care’ OR ’preconception’ OR ’periconception’ OR ’preconception care’

[2] ’pregnancy interval*’ OR ’birth spacing’ OR ’first birth interval’ OR ’interpregnancy interval’ OR ’intergenesic interval’ OR ’pregnancy spacing’ OR ’family planning’ OR ’delaying childbearing’ OR ’delaying pregnancy’ OR ’delay pregnancy’ OR ’reproductive life plan*’

OR with: ab.ti

[3] ’reproductive aged women’ OR ’teen*’ OR ’adoles*’ OR ’girl*’

[1] AND [2] AND [3]

[1] AND [2]

Embase: Iron/Folic Acid

[1] ’prepregnancy care’ OR ’preconception’ OR ’periconception’ OR ’preconception care’

[2] ’folic acid’/exp OR ’iron folic acid’ OR ’folic acid supplementation’ OR ’iron folic acid supplementation’

[3] "reproductive aged women" OR "teen*" OR "adolesc*" OR "girl" OR "pubescent girl*" OR "menstruating girl*" OR "menstruating women"

[1] AND [2] AND [3]

CINAHL: Delay/Interval

[1] (MM "Prepregnancy Care") OR "preconception" OR "preconception care" OR "periconception"

[2] (MM "Birth Intervals") OR "birth intervals" OR "birth spacing" OR “pregnancy interval” OR "interpregnancy interval" OR "pregnancy spacing" OR "delaying pregnancy" OR "delaying childbearing" OR "delay pregnancy" OR "reproductive life plan" = 484

[3] "reproductive aged women" OR (MH "Maternal Age 35 and Over") OR (MH "Adolescent Mothers") OR (MH "Expectant Mothers") OR (MH "Multiparas") OR (MH "Primiparas") OR (MH "Pregnancy in Adolescence") OR "teenager" OR (MH "Adolescence") OR "girl"

[1] AND [2] AND [3]

[1] AND [2]

CINAHL: Iron/Folic Acid

[1] (MM "Prepregnancy Care") OR "preconception" OR "preconception care" OR "periconception"

[2] (MH "Folic Acid") OR "iron folic acid" OR "folic acid supplementation" OR "folic acid supplement" OR "iron folic acid supplement"

[3] "reproductive aged women" OR "teenager" OR (MH "Adolescence") OR "girl" (MH "Puberty") OR (MH "Menarche") OR "pubescent" OR (MH "Puberty") OR (MH "Menarche") OR "pubescent girl*" OR "menstruating girl*" OR "menstruating women"

[1] AND [2] AND [3]

PsycINFO: Delay/Interval

[1] ("preconception care" or "prepregnancy care" or "preconception" or "periconception").af.

[2] exp family planning/ or birth control/ or condoms/ or delayed parenthood/ OR ("birth interval*" or "birth spacing" or "pregnancy interval" or "inter-pregnancy interval" or "pregnancy spacing" or "delaying pregnancy" or "delaying childbearing" or "delay pregnancy" or "delaying pregnancies" or "delay pregnancies" or "reproductive life plan").af. = 10,500

[3] adolescent pregnancy/ or adolescent mothers/ OR

("reproductive aged women" or "teenage girl*" or "adolescent girl*" or "women" or "girl*").af.

[1] AND [2] AND [3]

[1] AND [2] AND [3]

PsycINFO: iron/folic acid

[1] ("preconception care" or "prepregnancy care" or "preconception" or "periconception").af.

[2] folic acid/ OR "iron folic acid" OR "folic acid supplementation" OR "folic acid supplement" OR "iron folic acid supplement"

[3] ("reproductive aged women" or "teenage girl*" or "adolescent girl*" or "women" or "girl*" or "pubescent girl*" or "menstruating girl*" or "menstruating women" or "menarche").af.

[1] AND [2] AND [3]

[1] AND [2] AND [3] AND COUNTRY FILTERS

ERIC: Delay/Interval

[1] "preconception" OR "periconception" OR "pre-pregnancy" OR "preconception care" OR "prepregnancy care"

[2] "family planning" OR "reproductive plan*" OR "reproductive life plan*" OR "contraception" OR "birth control" OR "condom*" OR "birth interval" OR "birth spacing" OR "pregnancy interval" OR "intergenesic interval" OR "pregnancy spacing" OR "inter pregnancy interval" OR "sexual abstinence" OR "sterilisation" OR "delaying pregnanc*" OR "delay pregnanc*" OR "delay childbearing"

[3] "reproductive aged women" OR "adolescen*" OR "teen*" OR "teenage girl*" OR "girl*" OR "women"

[1] AND [2] AND [3]

[1] AND [2]

ERIC: Iron/Folic acid

[1] "preconception" OR "periconception" OR "pre-pregnancy" OR "preconception care" OR "prepregnancy care"

[2] "iron folic acid" OR "folic acid" OR "folate" OR "iron folic acid supplement*" OR "folic acid supplement*"

[3] = same as above

[1] AND [2]

[1] AND [2] AND [3]

Clinical Trials gov – Delay/Interval

"Family planning" OR "birth spacing" OR "pregnancy interval" AND "preconception" AND "adolescent"

Clinical trials gov – Iron/Folic Acid

"folic acid" OR "iron folic acid" AND "preconception" AND "adolescent"
