# Supplementary material for: Effects of Preconception Care and Periconception Interventions on Maternal Nutritional Status and Birth Outcomes in Low- and Middle-Income Countries: A Systematic Review
Source: Nutrients. 2020 Feb 26;12(3):606. doi: 10.3390/nu12030606 (PMC7146400; doi:10.3390/nu12030606)
Supplement: Supplementary file 1 [file nutrients-12-00606-s001.pdf]

Supplementary Table 1: Location the interventions were conducted, by intervention type

Location of intervention	Africa	Asia	South America	Central America	North America
**Delaying Pregnancy**	Cameroon<br>Ethiopia<br>Kenya<br>Malawi<br>Nigeria<br>Senegal<br>South Africa<br>Tanzania<br>Uganda<br>Zimbabwe	China<br>India<br>Vietnam	Chile	Mexico<br>Belize	
**Inter-pregnancy Intervals**		Bangladesh<br>China<br>India			
**Folic Acid Supplementation**		China		Cuba<br>Brazil	Honduras
**Iron-folic Acid Supplementation**	Mali<br>Tanzania	Bangladesh<br>India<br>Indonesia<br>Nepal			
